# Mutational Bias and Natural Selection Driving the Synonymous Codon Usage of Single-Exon Genes in Rice (*Oryza sativa* L.)

**DOI:** 10.1186/s12284-023-00627-2

**Published:** 2023-02-27

**Authors:** Huan Hu, Boran Dong, Xiaoji Fan, Meixia Wang, Tingzhang Wang, Qingpo Liu

**Affiliations:** 1 The Key Laboratory for Quality Improvement of Agricultural Products of Zhejiang Province, College of Advanced Agricultural Sciences, Zhejiang A & F University, Lin’an, Hangzhou, 311300 People’s Republic of China; 2 The Key Laboratory of Microbial Technology and Bioinformatics of Zhejiang Province, Hangzhou, 310012 People’s Republic of China

**Keywords:** Rice, Single-exon gene, Codon usage bias, Mutation bias, Natural selection

## Abstract

The relative abundance of single-exon genes (SEGs) in higher plants is perplexing. Uncovering the synonymous codon usage pattern of SEGs will benefit for further understanding their underlying evolutionary mechanism in plants. Using internal correspondence analysis (ICA), we reveal a significant difference in synonymous codon usage between SEGs and multiple-exon genes (MEGs) in rice. But the effect is weak, accounting for only 2.61% of the total codon usage variability. SEGs and MEGs contain remarkably different base compositions, and are under clearly differential selective constraints, with the former having higher GC content, and evolving relatively faster during evolution. In the group of SEGs, the variability in synonymous codon usage among genes is partially due to the variations in GC content, gene function, and gene expression level, which accounts for 22.03%, 5.99%, and 3.32% of the total codon usage variability, respectively. Therefore, mutational bias and natural selection should work on affecting the synonymous codon usage of SEGs in rice. These findings may deepen our knowledge for the mechanisms of origination, differentiation and regulation of SEGs in plants.

**Supplementary Information:**

The online version contains supplementary material available at 10.1186/s12284-023-00627-2.

## Background

Synonymous codon usage bias that is characterized by the preferential usage of one or several synonymous codons in the process of protein-coding genes, is ubiquitous in eukaryotes and prokaryotes (Quax et al. 2015). Interestingly, significant difference in synonymous codon usage is present not only among different species (Chakraborty et al. 2020), but even between different types of genes within the same species (Liu 2012). For instance, different types of tissue-specific genes have distinct codon usage in rice (Liu 2012). As reported, a number of factors are involved in shaping the codon usage bias, such as mutational pressure, environmental choice, gene length, tRNA richness, and organ specificity (Holmquist and Filipski 1994; Moriyama 1997; Chen 2013). Such bias in codon usage could broadly influence mRNA expression level through transcription in a translation-independent manner (Zhao et al. 2021).

In eukaryotes, protein-coding genes can be divided into two groups, single-exon genes (SEGs) and multiple-exon genes (MEGs), based on their exon numbers. Compared with eukaryotes, prokaryotes contain greatly higher proportion of SEGs in the genomes (Sakharkar et al. 2004). However, most eukaryotic SEGs are present only in eukaryotes. In rice and *Arabidopsis*, more than 68% and 77% of SEGs, respectively, have no homologous genes in prokaryotes (Jain et al. 2008). It is thus intriguing to investigate the evolutionary mechanism of SEGs in eukaryotes. Recently, a great progress has been made for the identification and characterization of SEGs in eukaryotic genomes (Jain et al. 2008; Yan et al. 2014a), and several SEG databases including Genome SEGE (Sakharkar and Kangueane 2004), SinEx DB (Jorquera et al. 2016, 2021), PIGD (Yan et al. 2014b), RIGD (Chen et al. 2020), and IGDD (Yan et al. 2016) have been developed for further exploration the evolution of SEGs in regards to the synonymous codon usage.

Eukaryotic SEGs may play crucial roles in regulation of important biological processes (Dong et al. 2019; Yuan et al. 2019), although they are typically expressed at lower levels and in a tissue-specific manner (Shabalina et al. 2010; Grzybowska 2012). Hence, exploring the codon usage pattern of SEGs could be useful for further uncovering their function and evolution. However, whether there is significant codon usage variation between SEGs and MEGs in plants has not been examined yet. In rice, the base composition of the genome is highly heterogeneous (Liu 2012), which might play a determinant role in shaping the codon usage of SEGs and MEGs. In addition, if different evolutionary forces impose on SEGs and MEGs remains elusive in rice.

In this study, the codon usage patterns of SEGs and MEGs were evaluated by adopting a multivariate method, internal correspondence analysis (Lobry and Chessel 2003; Sémon et al. 2006; Liu 2012). These analyses revealed a significant but weak difference in synonymous codon usage between SEGs and MEGs in rice. Notably, SEGs evolved significantly faster than MEGs. Both of the GC content, gene expression, and gene function were involved in determining the codon usage bias of SEGs. These findings shed new light on the understanding of the functional roles of SEGs in plants.

## Results

### Variation in Synonymous Codon Usage Between Single-Exon Genes and Multiple-Exon Genes

A total of 11,281 single-exon genes (SEGs) and 40,904 multiple-exon genes (MEGs) (Additional file 1: Table S1) were fed to CodonW and custom python programs to explore their synonymous codon usage pattern in rice. Here, two effective indicators, the Effective Number of Codons (ENC) and the Codon Adaptation Index (CAI) were used to evaluate the codon usage bias of SEGs and MEGs. A smaller ENC value (Wright 1990) as well as a greater CAI value (Sharp and Li 1987) indicates a stronger codon usage bias. The average ENC value of SEGs is significantly smaller than that of MEGs (42.921 *vs.* 50.815, *p* < 2.2E^−16^; Table 1). Notably, 27.59% and 28.84% of SEGs have ENC values ≤ 35 and > 50, respectively; while the percentage of MEGs having ENC values ≤ 35 and > 50, is 6.82% and 66.52%, respectively (Additional file 2: Table S2). In contrast, the average CAI value of SEGs is significantly greater than that of MEGs (0.395 *vs.* 0.234, *p* < 2.2E^−16^; Table 1). Furthermore, the ENC values of SEGs and MEGs were both significantly negatively correlated with their CAI values (*Spearman*’s correlation coefficient, *r* =  − 0.879, *p* < 0.01; and *r* =  − 0.483, *p* < 0.01). These observations indicate that strong variation in synonymous codon usage should have occurred between SEGs and MEGs in rice, with the former having apparently stronger synonymous codon usage bias.

**Table 1:** Comparison of ENC and CAI values of single-exon genes, multiple-exon genes, and multiple-exon gene samplings in rice

Gene type	ENC	CAI
Single-exon gene	42.921 ± 9.592 b	0.395 ± 0.228 a
Multiple-exon gene	50.815 ± 7.800 a	0.234 ± 0.172 b
Multiple-exon gene sampling	50.738 ± 7.852 a	0.236 ± 0.173 b

Data are reported as means ± SD

Within a column, mean values followed by different letters indicate significant difference at the 0.05 level (*p* < 0.05)

To avoid any analysis bias caused by the difference in the number of SEGs and MEGs (11,281 *vs.* 40,904), we conducted 1000 samplings by randomly choosing the same number of genes (11,281) from the group of MEGs as that of SEGs, and calculated their ENC and CAI values accordingly. As expected, the average ENC and CAI values calculated by MEG samplings are not clearly different from that obtained by the whole dataset of MEGs (50.738 *vs.* 50.815, *p* > 0.05; and 0.236 *vs.* 0.234, *p* > 0.05; Table 1); while those values are significantly greater and smaller than that of SEGs (50.738 *vs.* 42.921, *p* < 2.2E^−16^; and 0.236 *vs.* 0.395, *p* < 2.2E^−16^; Table 1). The results indicate that SEGs and MEGs are highly differential in synonymous codon usage, which is regardless of their difference in gene numbers.

**Fig. 1:**
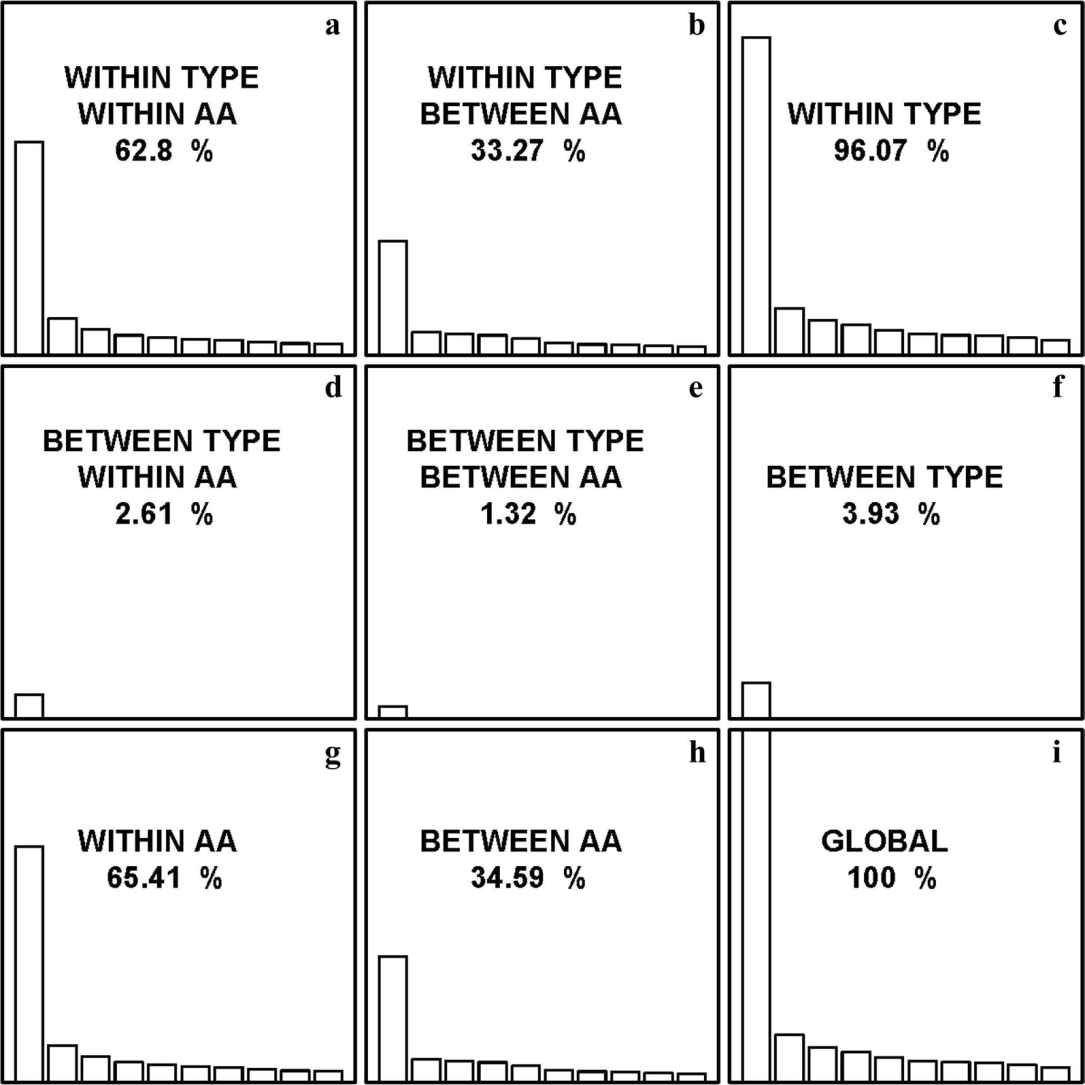
Internal correspondence analysis of single-exon genes and multiple-exon genes in rice. The total codon usage variability is decomposed into the synonymous codon usage (within-AA) variability (**a**, **d**, **g**), amino acid usage (between-AA) variability (**b**, **e**, **h**), and variability of within (**a**, **b**, **c**) and between gene types (**d**, **e**, **f**). The performance of ICA yields nine elementary analyses (**a–i**). In each peculiar analysis, the contribution to the total codon usage variation is indicated, where only the first 10 eigenvalues are represented for comparison

The internal correspondence analysis (ICA) was performed to further investigate the variability of synonymous codon usage between SEGs and MEGs. In ICA, the total codon usage variability was decomposed into four parts of codon usage variability, including the amino acid usage (between-AA) variability, synonymous codon usage (within-AA) variability, and variability of between or within different types of rice genes. From Fig. 1, it is evident that the variability of within different gene types is the major determinant to the total codon usage variability (96.07%; Fig. 1c); Comparatively, only 3.93% of the total codon usage variability is due to the variability of between different gene types (Fig. 1f). On the other hand, 65.41% and 34.59% of the total codon usage variability are attributed to the within-AA variability and between-AA variability (Fig. 1g and h). Compared with other sources of variability, the effect of synonymous codon usage variability between different gene types is much small, accounting for only 2.61% of the total codon usage variability (Fig. 1d).

To test whether the observed small proportion of variability in synonymous codon usage between SEGs and MEGs is caused just by chance, two kinds of permutations were performed, and ICA was repeated accordingly. The first permutation is to randomly choose 1000 samplings from the MEGs, with each sampling having the same gene number as that of SEGs (11,281). The average proportion of the variability of synonymous codon usage between different gene types obtained from 1000 independent samplings (3.20 ± 0.072%) is even greater than the observed value (2.61%). However, when randomly assigning rice genes into the SEG and MEG groups by 1000 independent permutations, the observed value (2.61%) is significantly greater than that obtained by chance (0.0002% ± 0.000%, *p* < 0.001). These results imply that SEGs and MEGs are truly distinct in synonymous codon usage.

### Base Compositional Bias and the Variability in Synonymous Codon Usage of Single-Exon Genes and Multiple-Exon Genes

**Fig. 2:**
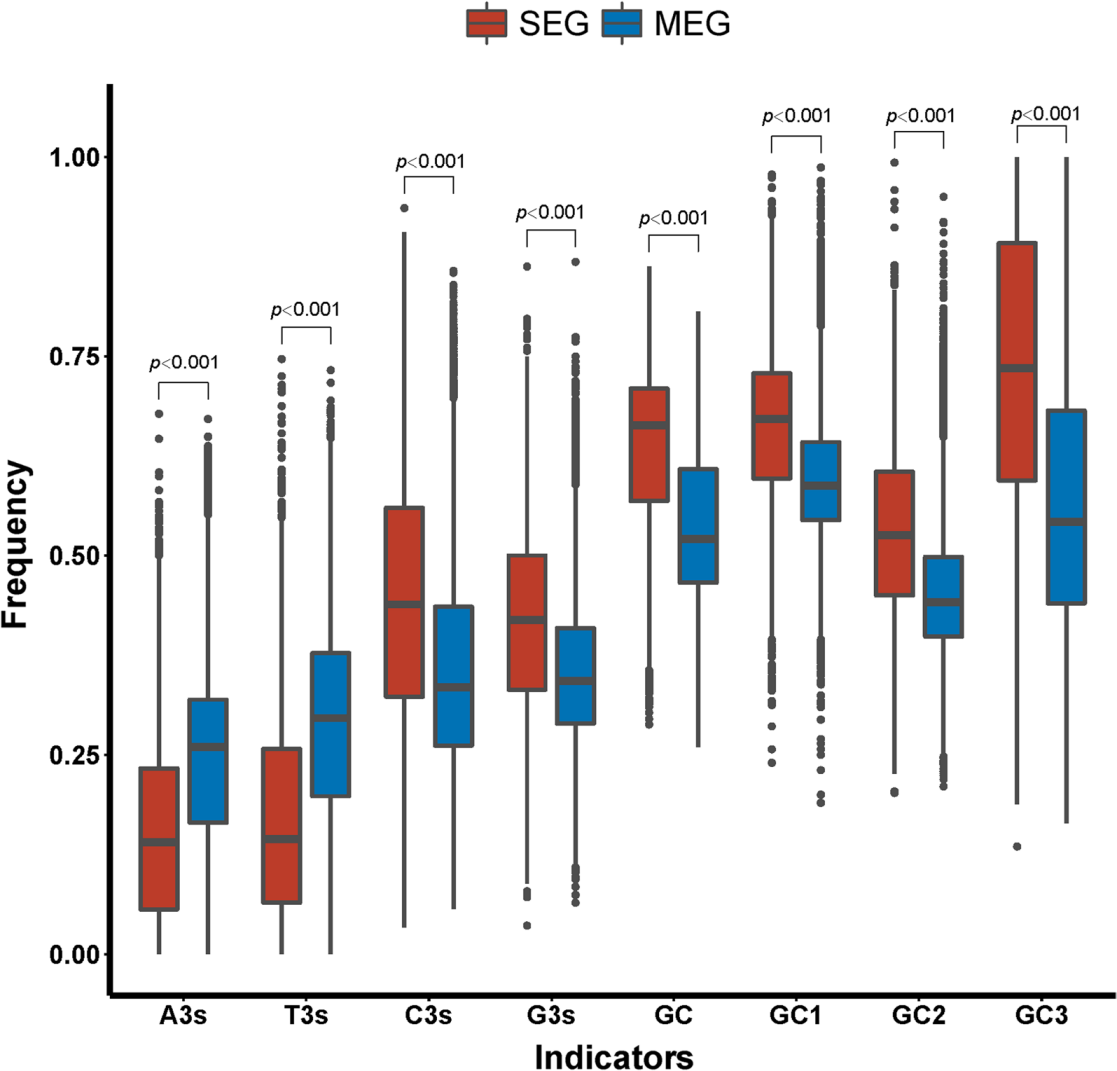
Comparison of base composition of coding sequences of single-exon genes and multiple-exon genes in rice. Here, A3s, T3s, C3s, or G3s is the frequency that codons have an A, T, C, or G at their synonymous third position, relative to the amino acids that could have a synonym with A, T, C, or G in the synonymous third codon position. GC1, GC2, or GC3 is the percentage of GC content at the first, second, and third codon position, respectively

The nucleotide compositions of coding sequences (CDS) are clearly differential between SEGs and MEGs (Fig. 2). SEGs contain significantly lower frequency of A3s and T3s, but greatly higher frequency of C3s and G3s, as compared with MEGs (Fig. 2), where A3s, T3s, C3s, or G3s is the frequency that codons have an A, T, C, or G at their synonymous third position, relative to the amino acids that could have a synonym with A, T, C, or G in the synonymous third codon position (Peden 1999). Moreover, both the global GC content and the percentage of GC content of the three codon positions are significantly greater in SEGs than that in MEGs (*p* < 0.001; Fig. 2). In SEGs, the third codon position (GC3) has the highest GC content, and followed by the first (GC1) and second (GC2) codon positions; whereas in MEGs, the first and second codon positions (GC1 and GC2) have the highest and lowest GC content, respectively. Thus, the largest difference in GC content is at the third codon position (GC3) of SEGs and MEGs (Fig. 2).

**Fig. 3:**
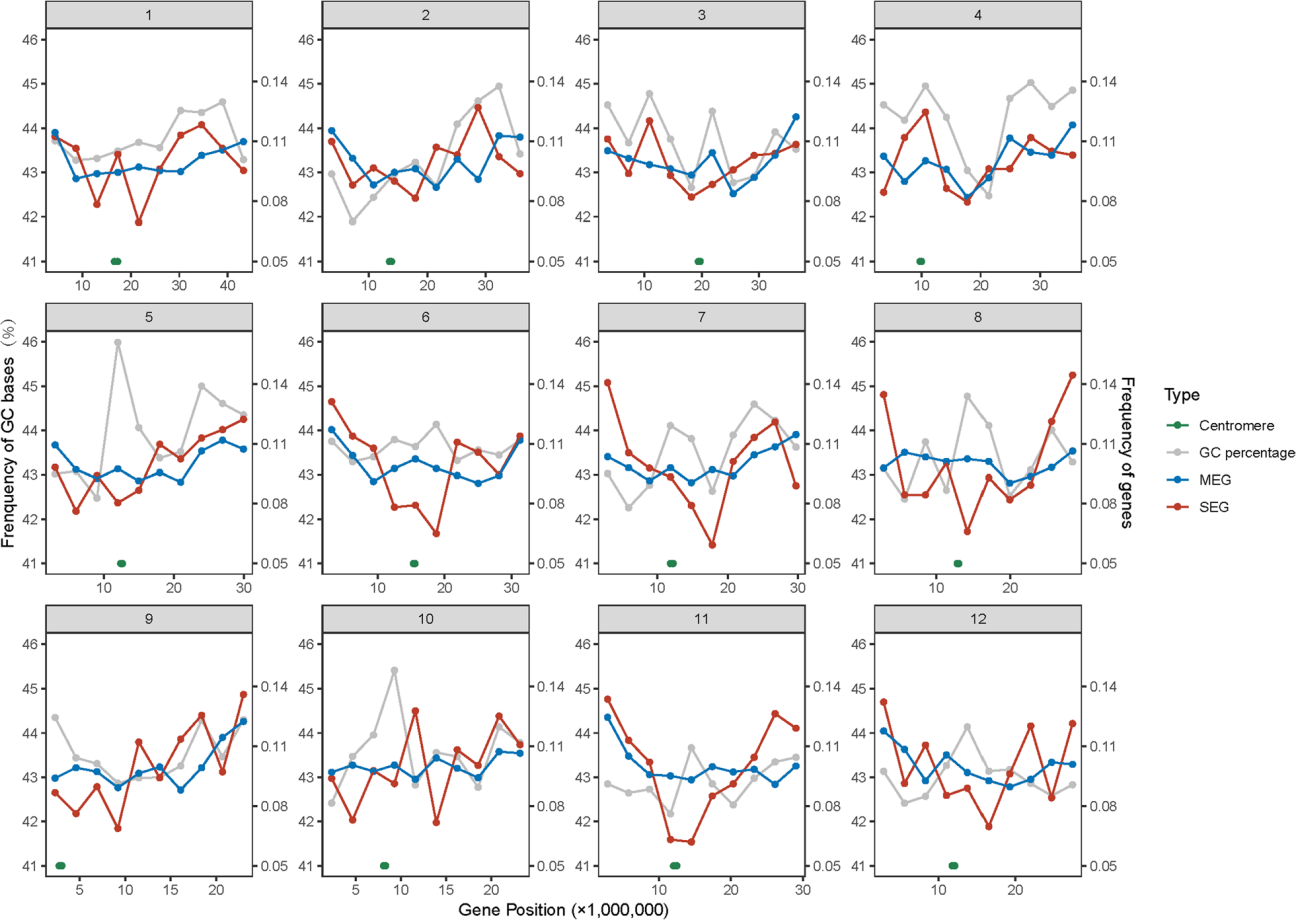
Distribution of single-exon genes, multiple-exon genes and GC content along each of the twelve chromosomes in rice. Each of the chromosome is divided into ten equal sequence regions. The percentage of GC content, and the numbers of single-exon genes and multiple-exon genes are calculated and counted accordingly

To discern whether the higher GC3 and GC content of SEGs may be caused coincidently by the background GC content of their surrounding genomic regions, the distribution and characteristics of SEGs and MEGs along rice chromosomes were examined. To this end, each of the twelve chromosomes was equally separated into ten sequence regions, and the GC content and the number of SEGs and MEGs in each region were calculated and counted accordingly. It is clear that both the GC content and the numbers of SEGs and MEGs are unevenly distributed along rice chromosomes (Fig. 3). Compare with MEGs, the SEG numbers are extensively differential among sequence regions. SEGs tend to enrich towards the ends of chromosomes, but avoid to accumulate in the centromere regions, especially on chromosomes 8 and 11 (Fig. 3). Notably, the regional GC contents are significantly positively correlated with the numbers of SEGs and MEGs (*Spearman*’s correlation coefficient, *r* = 0.262, *p* = 3.895E^−3^; and *r* = 0.312, *p* = 5.127E^−4^) at the whole genome-wide level. Nonetheless, the correlations between the regional GC contents and the numbers of SEGs and MEGs are not significant at the chromosomal level, with two exceptions where the regional GC contents are significantly positively correlated with the numbers of MEGs on chromosomes 4 and 10 (*Spearman*’s correlation coefficient, *r* = 0.769, *p* = 9.222E^−3^; and *r* = 0.705, *p* = 2.274E^−2^). In addition, the average GC content of 200-bp noncoding sequences flanking every side of each SEG and MEG (GCf) was calculated. The CAI values of SEGs and MEGs are significantly positively correlated with GCf content (*Spearman*’s correlation coefficient, *r* = 0.117, *p* < 0.001; and *r* = 0.229, *p* < 0.001). These observations are indicative of stronger effect of environmental GC content on the codon usage of MEGs in rice.

**Fig. 4:**
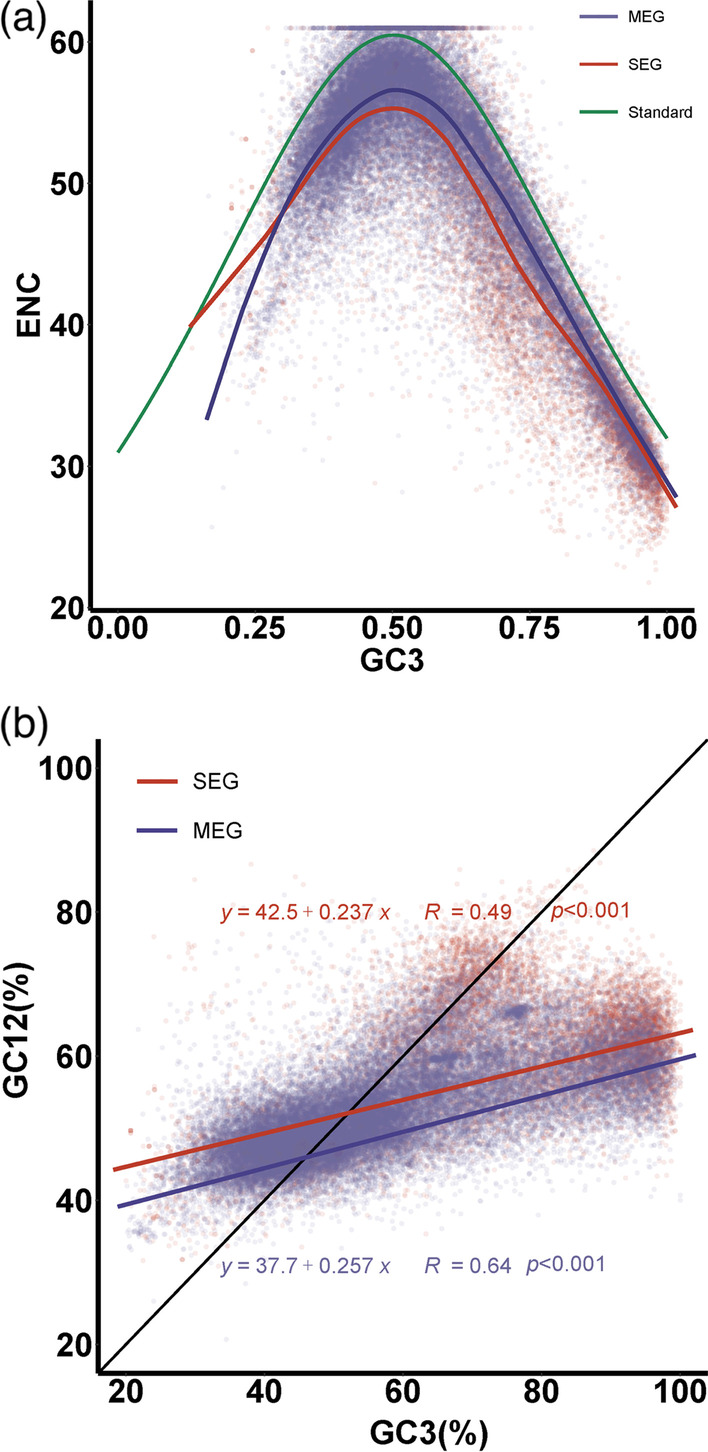
ENC plot (ENC *vs.* GC3) and neutrality plot (GC12 *vs.* GC3) analyses of single-exon genes and multiple-exon genes in rice. **a** ENC plot analysis. The theoretical curve (green) represents the expected relationship between ENC and GC3 values. The simulated polynomial regression lines are shown in red and blue for the SEGs and MEGs (under the theoretical line), respectively. **b** Neutrality plot analysis. The theoretical line (black) represents the expected relationship between GC12 and GC3 values. The simulated regression lines are shown in red and blue for the SEGs and MEGs, respectively

Whether the base compositional preference is primarily responsible for the observed significant difference in synonymous codon usage between SEGs and MEGs? To answer this, an ENC plot analysis of codon usage and nucleotide content (ENC *vs.* GC3) was conducted, where the nucleotide composition at the third codon position would be the sole determinant factor of codon usage, if the gene points of ENC against GC3 values fall on the theoretical curve (Novembre 2002). It is clear that most of the SEGs and MEGs fall below the expected curve (green; Fig. 4a). However, the observed ENC values of MEGs track the theoretical curve closely (blue), while the simulated polynomial regression line of ENC on GC3s for SEGs (red) is relatively far away from the expected line (Fig. 4a). On the other hand, the Neutrality plot (GC12 *vs.* GC3) was performed to further explore the relationship between base compositional bias and codon usage variation. If the points of GC12, the average value of GC1 and GC2, against GC3 values fall on the diagonal standard line, mutational bias would be the sole factor in shaping the synonymous codon usage (Sueoka 2001). The simulated regression lines of SEGs (red) and MEGs (blue) do apparently not overlap with the expected line (black; Fig. 4b). The GC12 values are significantly positively correlated with GC3 values in both SEGs and MEGs (*Spearman*’s correlation coefficient, *r* = 0.49, *p* < 0.001; and *r* = 0.64, *p* < 0.001; Fig. 4b). Furthermore, the slope coefficients of the simulated regression lines of SEGs and MEGs are significantly different (*p* < 2.2E^−16^). These significant correlations between GC12 and GC3 values suggest the strong base compositional bias in SEGs and MEGs, with the latter having relatively higher mutational bias or lower conservation of GC content level among genes. According to the results of ENC plot (Novembre 2002) and Neutrality plot analyses (He et al. 2020), it is reasonable to infer that both mutational bias and natural selection work on the codon usage bias of SEGs and MEGs, but relatively stronger mutational pressure should impose on the codon usage of MEGs in rice.

### Selective Constraints and Functional Differentiation of Single-Exon Genes and Multiple-Exon Genes

**Fig. 5:**
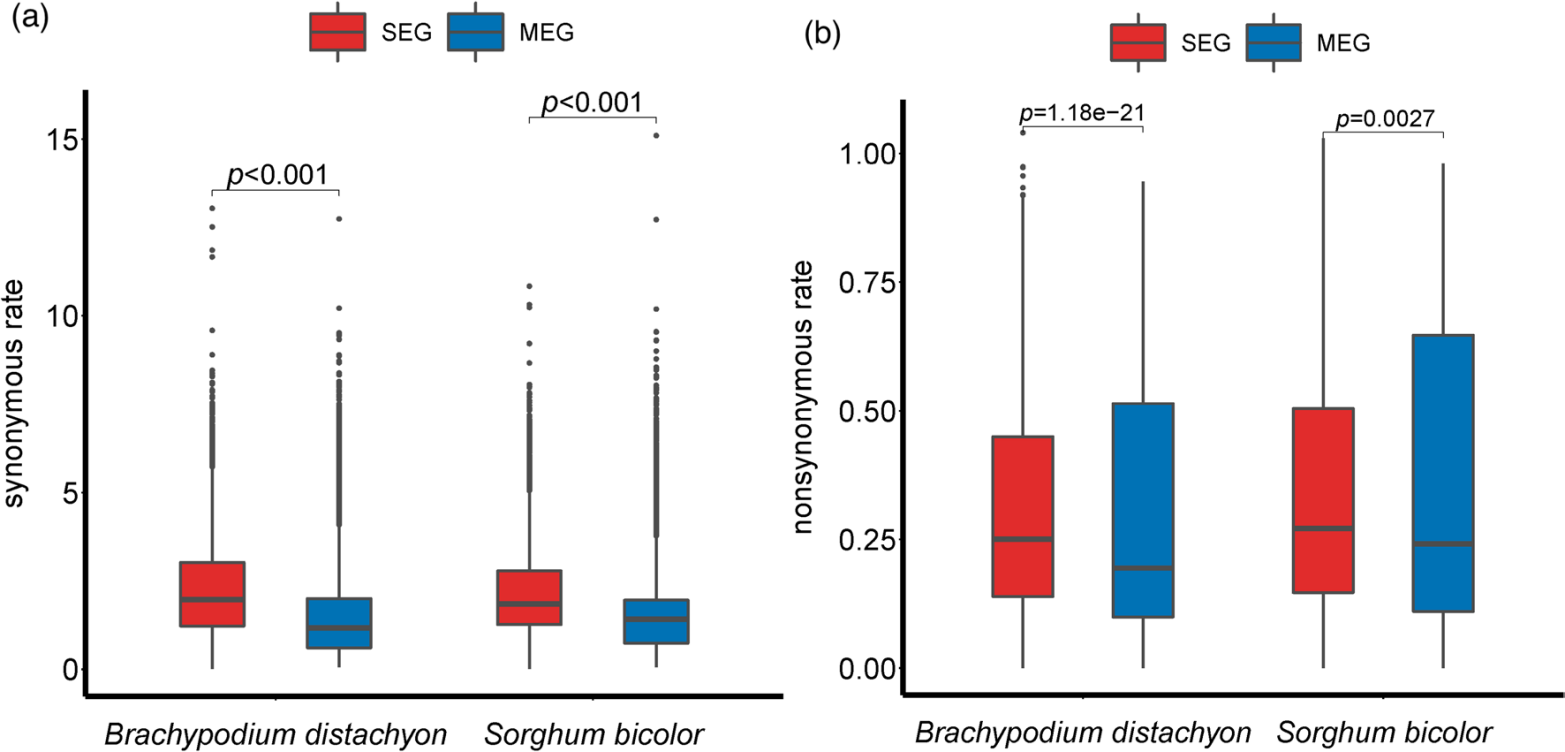
Comparison of the synonymous (**a**) and non-synonymous substitution rates (**b**) between rice and *Brachypodium distachyon*, and *Sorghum bicolor* orthologous gene pairs of single-exon genes and multiple-exon genes in rice

Cusack et al. (2011) revealed that SEGs tend to select robust codons to prevent the mis-transcription under selection pressure. Thus, it is intriguing to test whether differential selective constraints impose on SEGs and MEGs in rice. The synonymous (Ks) and non-synonymous substitution rate (Ka) between rice and *B. distachyon*, and *S. bicolor* orthologous gene pairs were calculated, and subjected to investigating the evolutionary rate of SEGs and MEGs. At the first glance, both SEGs and MEGs should be under strong purifying selection, as reflected from their small Ka/Ks ratios that are significantly less than 1.0 (0.165 ± 0.121 and 0.183 ± 0.138 for SEGs, *p* < 0.001; and 0.224 ± 0.142 and 0.238 ± 0.156 for MEGs, *p* < 0.001, respectively). However, the comparisons of Ks and Ka rate values of orthologous gene pairs show that SEGs have obviously higher mutational rate, as reflected from their significantly higher Ks and Ka rates when compared with MEGs (*p* < 0.001 and *p* < 0.001for Ks; and *p* = 1.18E^−21^ and *p* = 2.7E^−3^ for Ka; Fig. 5). The results indicate that SEGs must evolve remarkably faster than MEGs during evolution, although the two types of genes are all under strong selective pressures.

**Fig. 6:**
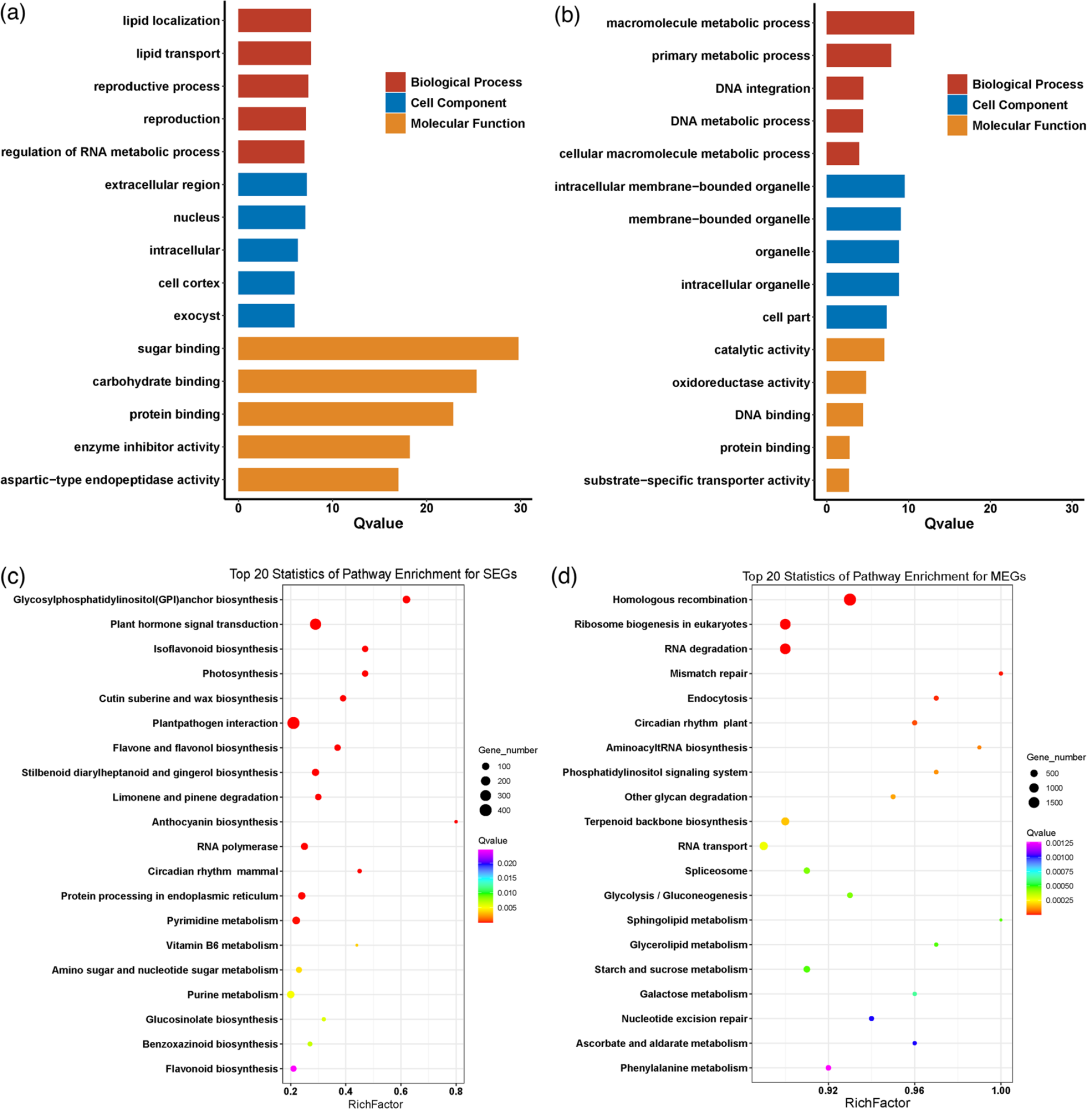
Gene ontology (GO) and KEGG pathway enrichment analyses of single-exon genes and multiple-exon genes in rice. **a**–**b** GO enrichment analysis of SEGs (**a**) and MEGs (**b**), which is performed by adopting the singular enrichment analysis (SEA) implemented in AgriGO v2.0. (**c**–**d**) Pathway enrichment analysis of SEGs (**c**) and MEGs (**d**), which is conducted by searching against the KEGG database

The gene ontology (GO) analysis was carried out to gain an insight into the functional enrichment of SEGs and MEGs. As shown in Fig. 6a, SEGs mainly function in sugar-, carbohydrate-, and protein-binding, and participate in the biological processes of regulation of lipid localization, lipid transport, and reproduction, etc. Comparatively, MEGs usually have catalytic and oxidoreductase activity, and participate in the macromolecule and primary metabolic processes (Fig. 6b). Furthermore, the KEGG database was searched to explore the pathway enrichment of SEGs and MEGs. SEGs are abundantly related to the following pathways, including the “Glycosylphosphatidylinositol (GPI) anchor biosynthesis”, “Plant hormone signal transduction”, “Isoflavonoid biosynthesis”, “Photosynthesis”, “Cutin suberine and wax biosynthesis”, and “Plant-pathogen interaction”, etc.(Fig. 6c). On contrast, the enriched pathways for MEGs highly concentrate on the “Homologous recombination”, “Ribosome biogenesis in eukaryotes”, “RNA degradation”, “Mismatch repair”, etc.(Fig. 6d). It seems that functional divergence should have occurred between SEGs and MEGs in rice.

### Dissection of Factors Affecting the Synonymous Codon Usage of Single-Exon Genes

The ICA, ENC plot, and Neutrality plot analyses present the general measure of potential factors affecting the codon usage of SEGs in rice. To better understand the contribution of different factors to the codon usage variation of SEGs, extra ICA was separately performed by dividing the SEGs into different groups, based on their GC content, gene expression level, and gene function.

#### GC3 Content Variation and Codon Usage Bias

**Fig. 7:**
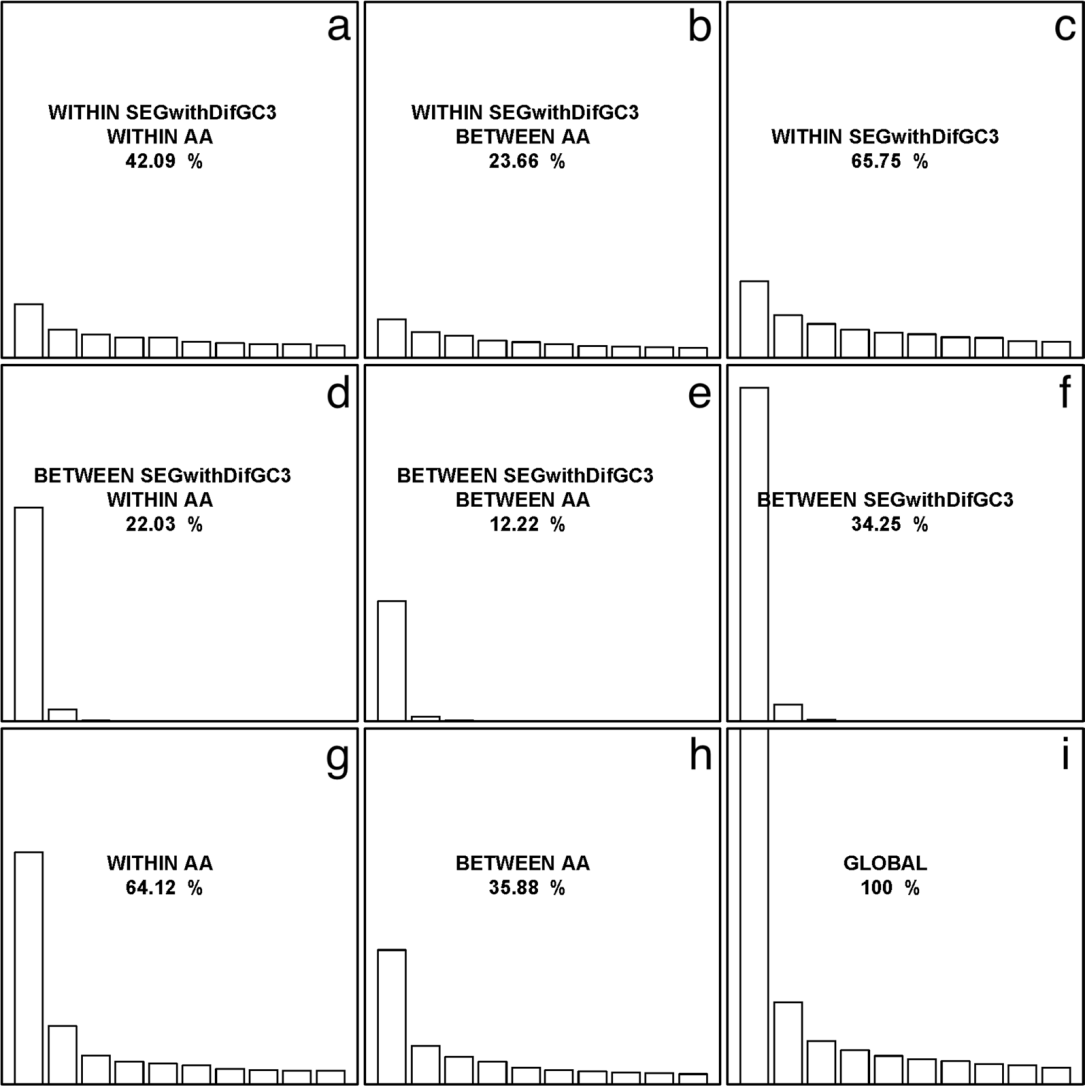
Internal correspondence analysis of single-exon genes with different GC3 content in rice. The total codon usage variability is decomposed into the synonymous codon usage variability (**a**, **d**, **g**), amino acid usage variability (**b**, **e**, **h**), and variability of within (**a**, **b**, **c**) and between different GC3 content groups (**d**, **e**, **f**). The performance of ICA yields nine elementary analyses (**a–i**). In each peculiar analysis, the contribution to the total codon usage variability is indicated, where only the first 10 eigenvalues are represented for comparison

The 11,281 SEGs were divided into five groups (≥ 0.8, 0.8–0.7, 0.7–0.6, 0.6–0.5, < 0.5) based on their GC3 content, consisting of 4499, 1916, 1984, 1174, and 1708 genes in the corresponding groups. The result of ICA shows that 65.75% and 34.25% of the total codon usage variability are due to the variability of within and between different GC3 content groups, respectively (Fig. 7c, f); On the other hand, 64.12% and 35.88% of the total codon usage variability can be owing to the synonymous codon usage (within-AA) variability (Fig. 7g) and amino acid usage (between-AA) variability (Fig. 7h), where the variability in synonymous codon usage between different GC3 content groups accounts for 22.03% of the total codon usage variability in SEGs (Fig. 7d). Particularly, the ENC and CAI values of SEGs are strongly negatively and positively correlated with GC3 content (*Spearman*’s correlation coefficient, *r* =  − 0.881, *p* < 0.001; and *r* = 0.996, *p* < 0.001). Thus, it infers strongly that mutational bias should play an essential role in determining synonymous codon usage of SEGs in rice.

#### Gene Expression Level and Codon Usage Bias

To examine the effect of gene expression variation on the synonymous codon usage of SEGs, the 11,281 SEGs were divided into five groups (≥ 2.0, 2.0–0.65, 0.65–0.3, 0.3–0.13, < 0.13), based on gene expression level assessed by RNA-Seq (He et al. 2010), with each group having the same number of gene as that in each of the five GC3 content groups. The performance of ICA reveals that the synonymous codon usage (within-AA) variability accounts for 64.33% of the total codon usage variability (Additional file 3: Fig. S1). However, only 3.32% of the total codon usage variability can be explained by the variability in synonymous codon usage between different gene expression groups (Additional file 3: Fig. S1).

Whether gene expression shows similar relationship with GC3 content and GC content of the flanking noncoding sequence region, serves as an effective way to test the effect of translational selection on the synonymous codon usage bias of SEGs. If the two relationships are similar, no selection would be expected (Qiu et al. 2011). The gene expression level is significantly positively correlated with GC3, but not with GCf content of SEGs (*Spearman*’s correlation coefficient, *r* = 0.501, *p* < 0.001; and *r* = -0.017, *p* = 7.268E^−2^). Notwithstanding, there is a significant but weak positive correlation between GC3 and GCf content in SEGs (*Spearman*’s correlation coefficient, *r* = 0.027, *p* = 4.667E^−3^). The results imply that translational selection is involved in shaping the synonymous codon usage of SEGs, but the effect would be relatively weak.

#### Gene Function and Codon Usage Bias

Based on the functional enrichment analysis (Fig. 6a), the SEGs belonging to the top five enriched functional groups were used to perform ICA. Only 5.99% of the total codon usage variability is due to the variability in synonymous codon usage between different functional groups (Additional file 3: Fig. S2), indicating that the gene functional bias indeed affects the codon usage variation of SEGs in rice.

## Discussion

Jorquera et al. (2021) evidenced that the percent of SEGs is about 10% and 20% in the genome of animals and plants. In this study, a total of 11,281 SEGs that accounts for 21.6% of the rice genes were identified, with the percentage of SEGs being similar as that reported in other plants (Jorquera et al. 2021). However, Sakharkar et al. (2004) reported that there were 12.3% of SEGs in *Homo sapiens*. Jain et al. (2008), and Liu et al. (2021) revealed 19.9% and 24.5% of SEGs in the rice genome, respectively. The incomplete annotation of the genome used by Sakharkar et al. (2004) should be responsible for the inconsistency in the proportion of SEGs in *H. sapiens*. As for the inconsistency between Jain et al. (2008), Liu et al. (2021) and the present study, the prediction pipeline as well as the screening filtration for rice SEGs are clearly different, which should be responsible for the difference in the identified SEG numbers in rice.

The SEG proportion decreases with the total gene count in the genomes, and prokaryotes and basal eukaryotes usually have higher proportion of SEGs in their genomes (Sakharkar et al. 2004). In *Encephalitozoon cuniculi* and *Saccharomyces cerevisiae*, the proportion of SEGs can reach up to 97.7% and 92.5%, respectively (Sakharkar et al. 2004). Comparatively, the percent of SEGs in most mammals is around 10%, with 11.9% in pigs, 9.7% in horses, 10.4% in chimpanzees, and 8.9% in humans (Jorquera et al. 2016; 2021). It is reasonable to infer that the significantly higher proportion of SEGs in unicellular prokaryotes must be beneficial to their rapid proliferation with short generation times (Sakharkar et al. 2004).

During the past decades, amounts of SEGs have been identified in eukaryotes (Sakharkar et al. 2004; Jain et al. 2008; Shabalina et al. 2010; Yan et al. 2014a, b; Jorquera et al. 2021). However, a large fraction of eukaryotic SEGs lack homology with prokaryotic genes (Sakharkar et al. 2004; Jain et al. 2008; Yan et al. 2014a, b), suggestive of the evolutionary origination of SEGs by retroposition (Sakharkar et al. 2004; Wang et al. 2006; Yan et al. 2014a, b). In this study, the paralogous gene pairs of SEGs and MEGs were identified by searching the PlantDGD database (Qiao et al. 2019). Totally, 581 SEGs that probably arose by retroposition have paralogs of MEGs, of which 373 and 208 SEGs might be generated via tandem and segmental duplications (Additional file 4: Table S3). To clarify whether the retroposition mechanism affects the divergence of synonymous codon usage of SEGs and MEGs, the 581 SEG/MEG paralogous genes were excluded from the dataset, and the ICA was conducted accordingly (Additional file 3: Fig. S3). However, the effect of synonymous codon usage variability between different gene types (2.5%) is slightly smaller than that obtained using the whole dataset (2.61%), indicative of the relatively weaker effect of retroposition mechanism on the codon usage bias of SEGs and MEGs in rice.

Neutral evolution and natural selection are considered as the major determinants in shaping the synonymous codon usage of a set of specific genes and even a given genome (Iriarte et al. 2021). In rice, the synonymous codon usage bias of tissue-specific genes is mainly due to the mutational bias and natural selection (Liu 2012). However, in some eukaryotes, translational selection is the major factor in determining the codon usage bias (Qiu et al. 2011). In this study, we uncovered a significant but weak synonymous codon usage variation between SEGs and MEGs in rice, which might primarily arise from base compositional mutation bias. In particular, stronger effect of environmental GC content on shaping the synonymous codon usage pattern is evidenced in MEGs, as compared with that in SEGs. Further, given that SEGs and MEGs are under differential selective constraints, natural selection for gene expression and function should contribute to the codon usage bias of SEGs and MEGs too. Besides, SEGs and MEGs have significantly different CDS length (910.204 ± 742.991 *vs.* 1590.317 ± 1260.827 bp, *p* < 0.001), suggestive of the potential contribution of CDS length to their synonymous codon usage variability. Notably, in the group of SEGs, mutational bias may be essential for the synonymous codon usage variation among genes, and followed by gene function and gene expression level. However, natural selection works on shaping the codon usage bias of rice SEGs, but this effect is relatively weak.

Without undergoing the process of intron splicing, intronless genes are usually rapidly regulated and respond to stress conditions (Jeffares et al. 2008). In maize, a large number of intronless genes involved in immune response facilitate maize plants to quickly respond to biotic and abiotic stresses (Yan et al. 2014a, b). Similarly, the repeated arrangement of SEGs in the dinoflagellate genome is significantly beneficial to its adaptation to the cold environment in polar regions (Stephens et al. 2020). In this study, SEGs are found to evolve relatively faster than MEGs, and amounts of SEGs are enriched in the plant-pathogen interaction pathway, implying their involvement in response to environmental stresses.

## Materials and Methods

### Sequence Data

The rice (*Oryza sativa* L. *japonica*) gene sequences and genomic annotations were downloaded from the rice genomic resource (MSU pseudomolecule v7.0; ftp://ftp.plantbiology.msu.edu/) (Ouyang et al. 2007). The protein-coding sequences of *Brachypodium distachyon* and *Sorghum bicolor* were retrieved from the Phytozome database (v13.0; http://www.phytozome.net/) (Goodstein et al. 2012). The RNA-Seq data derived from rice shoots at the four-leaf seedling stage (He et al. 2010) was downloaded from the MSU database (http://rice.uga.edu/pub/data/Eukaryotic_

Projects/o_sativa/annotation_dbs/pseudomolecules/version_7.0/all.dir/final_rice_v7_expression_matrix_48columns.txt).

### Prediction and Classification of Single-Exon Genes and Multiple-Exon Genes

The gffread software (Pertea and Pertea 2020) was utilized to extract the intact gene information, on the basis of the annotation of rice genes. To avoid any analysis bias, the following genes were excluded from further analysis: (1) Genes having internal termination codon, or having abnormal start and/or stop codon; (2) Genes encoding tRNA, rRNA, or other noncoding RNAs (Jorquera et al. 2018); (3) Genes whose sequence length is less than 300 bp, as described previously (Liu et al. 2020); (4) Single exon isoforms (SEIs) generated from the alternative splicing of multiple-exon genes (MEGs) (Jorquera et al. 2018); (5) Genes located on unknown chromosomes, e.g. ChrSy and ChrUn. Accordingly, if a gene contains only one exon, it is considered as a single-exon gene (SEG); Otherwise, it is classified as a MEG. Notably, if a gene produces several alternative splicing variants, the longest CDS was used as the representative. Totally, 52,185 protein-coding genes were collected and subsequently divided into SEGs and MEGs (Additional file 1: Table S1).

### Measurement of Synonymous Codon Usage Bias

The Effective Number of Codons (ENC), an indicator that refers to the number of valid codons used in a gene, was calculated, yielding values ranging from 20 to 61. A smaller ENC value means a stronger codon usage bias (Wright 1990). The Codon Adaptation Index (CAI), which refers to the fitness coefficient when all the codons encode the protein using the optimal codon relative to this gene (Sharp and Li 1987), was also calculated to assess the codon usage bias. The CAI value ranges from 0 to 1.0, and a higher CAI value means a stronger codon usage bias (Sharp and Li 1987). The frequency of A3, T3, C3, and G3, where the usage of each nucleotide at synonymous third codon positions as a proportion of the maximum usage of that nucleotide could have without altering the amino acid composition (Peden 1999), and the frequency of G + C at the first, second, and third codon position (GC1, GC2, and GC3) were calculated after excluding the tryptophan, methionine, and three stop codons. In addition, the 200-bp noncoding sequences flanking every side of each SEG of MEG was extracted and the average GC content was calculated accordingly.

### ENC Plot Analysis

The ENC plot analysis was performed to uncover the factors affecting the codon usage bias by plotting the ENC and GC3 values of SEGs and MEGs. In this analysis, the standard curve between the expected ENC and GC3 values was described as the following formula (1) (Novembre 2002).

1 $${\mathrm{ENC}}_{\mathrm{exp}}=2+GC3s+\frac{29}{{GC3s}^{2 }+ {(1-GC3s)}^{2}}$$

If the true ENC value of each protein-coding sequence falls completely on the theoretical curve, or within a region closer to the theoretical curve, the GC3 may be the sole determinant of codon usage (Novembre 2002). While the point of ENC against GC3 value is under the standard curve, natural selection is supposed to be involved in the process of shaping the codon usage bias (Novembre 2002).

### Neutrality Plot Analysis

The neutrality plot analysis was performed to further analyze the main determinant factors for the codon usage of SEGs and MEGs by separately plotting their GC12 and GC3 values. Here, GC12 was calculated by the mean of GC1 and GC2. A higher correlation between GC12 and GC3 refers to much stronger effect of mutation pressure on codon usage. If the regression coefficient close to 1, it indicates that the codon usage bias might be mainly affected by mutation (Sueoka 2001). If there is no natural selection, the scatter points corresponding to GC12/GC3 will fall on the standard line with a slope of 1 (He et al. 2020).

### Internal Correspondence Analysis

Internal correspondence analysis (ICA) is an extension of correspondence analysis (Perrière and Thioulouse 2002). Previous studies demonstrated that ICA is an effective way in exploration of codon usage variation (Lobry and Chessel 2003; Sémon et al. 2006; Liu 2012). The SeqinR (Charif and Lobry 2007) and ade4 (Dray and Dufour 2007) packages implemented in R v4.1.1 (https://www.r-project.org/) were used to perform the ICA. In ICA, a codon usage table was constructed, and used to investigate the inter- and intra-type variability. According to ICA, the rows and columns will be split into blocks on the basis of the number of samples and amino acids. Based on this table, the total codon usage variability can be further decomposed into between-block and within-block variabilities, and the contribution of the variability in synonymous codon usage between different samples to the total codon usage variability will be inferred accordingly (Lobry and Chessel 2003; Sémon et al. 2006; Liu 2012).

### Identification of Orthologous Genes Pairs and Selective Constraint Analysis

The SEGs and MEGs were identified from *Brachypodium distachyon* and *Sorghum bicolor*, respectively, using the same method as described in rice. Then, the SEGs and MEGs identified in rice were separately used as query to search against the *B. distachyon* and *S. bicolor* SEG and MEG sequences to identify their orthologous gene pairs in each of the two species. The amino acid sequence alignments were carried out using MUSCLE (Edgar 2004) with default parameters, based on which the codon-alignments of CDS sequences were generated using PAL2NAL (Suyama et al. 2006). The programs ParaAT v2.0 (Zhang et al. 2012) and KaKs_Calculator v2.0 (Wang et al. 2010) were adopted to calculate the pair-wise synonymous (Ks) and non-synonymous (Ka) distance between orthologous genes of rice and *B. distachyon* and *S. bicolor*.

### Gene ontology (GO) and KEGG Pathway Enrichment Analysis

The singular enrichment analysis (SEA) was conducted using AgriGO v2.0 (Tian et al. 2017) to determine the biological functions of SEGs and MEGs. The KEGG database (Kanehisa et al. 2021) was employed to perform the pathway enrichment analysis of SEGs and MEGs in rice.

### Statistical Analysis

The calculation of ENC, CAI, A3, T3, G3, C3, GC, GC1, GC2, GC3, and CDS length was performed using CodonW v1.4.4 (Peden 1999) and custom python scripts. The performance of ICA, *Spearman* correlation, *Wilcoxon* signed rank test, and ANOVA analysis was all conducted using R v4.1.1.

## Supplementary Information

**Additional file 1.** **Table 1** The gene ID of single-exon genes and multiple-exon genes in rice.

**Additional file 2.** **Table 2** The dissection of ENC values of single-exon genes and multiple-exon genes in rice.

**Additional file 3.** **Figure 1.** Internal correspondence analysis of single-exon genes with different gene expression level in rice. The total codon usage variability is decomposed into the synonymous codon usage variability (a, d, g), amino acid usage variability (b, e, h), and variability of within (a, b, c) and between different gene expression groups (d, e, f). The performance of ICA yields nine elementary analyses (a-i). In each peculiar analysis, the contribution to the total codon usage variability is indicated, where only the first 10 eigenvalues are represented for comparison. **Figure 2.** Internal correspondence analysis of single-exon genes with different gene function in rice. The total codon usage variability is decomposed into the synonymous codon usage variability (a, d, g), amino acid usage variability (b, e, h), and variability of within (a, b, c) and between different gene function groups (d, e, f). The performance of ICA yields nine elementary analyses (a-i). In each peculiar analysis, the contribution to the total codon usage variability is indicated, where only the first 10 eigenvalues are represented for comparison. **Figure 3.** Internal correspondence analysis of single-exon genes and multiple-exon genes in rice. In this analysis, the 581 paralogous gene pairs of single-exon genes and multiple-exon genes are excluded from the dataset. The total codon usage variability is decomposed into the synonymous codon usage (within-AA) variability (a, d, g), amino acid usage (between-AA) variability (b, e, h), and variability of within (a, b, c) and between gene types (d, e, f). In each peculiar analysis, the contribution to the total codon usage variation is indicated, where only the first 10 eigenvalues are represented for comparison.

**Additional file 4.** **Table 3.** Paralogous gene pairs consisting of single-exon genes and multiple-exon genes generated via segmental and tandem duplications.

## Data Availability

All data supporting the conclusions of this article are available in this article and its online supplementary materials.
