# Supplementary material for: Mutational Bias and Natural Selection Driving the Synonymous Codon Usage of Single-Exon Genes in Rice (Oryza sativa L.)
Source: Rice (N Y). 2023 Feb 27;16:11. doi: 10.1186/s12284-023-00627-2 (PMC9971424; doi:10.1186/s12284-023-00627-2)
Supplement: Supplementary file 3 — Additional file 3. Figure 1. Internal correspondence analysis of single-exon genes with different gene expression level in rice. The total codon usage variability is decomposed into the synonymous codon usage variability (a, d, g), amino acid usage variability (b, e, h), and variability of within (a, b, c) and between different gene expression groups (d, e, f). The performance of ICA yields nine elementary analyses (a-i). In each peculiar analysis, the contribution to the total codon usage variability is indicated, where only the first 10 eigenvalues are represented for comparison. Figure 2. Internal correspondence analysis of single-exon genes with different gene function in rice. The total codon usage variability is decomposed into the synonymous codon usage variability (a, d, g), amino acid usage variability (b, e, h), and variability of within (a, b, c) and between different gene function groups (d, e, f). The performance of ICA yields nine elementary analyses (a-i). In each peculiar analysis, the contribution to the total codon usage variability is indicated, where only the first 10 eigenvalues are represented for comparison. Figure 3. Internal correspondence analysis of single-exon genes and multiple-exon genes in rice. In this analysis, the 581 paralogous gene pairs of single-exon genes and multiple-exon genes are excluded from the dataset. The total codon usage variability is decomposed into the synonymous codon usage (within-AA) variability (a, d, g), amino acid usage (between-AA) variability (b, e, h), and variability of within (a, b, c) and between gene types (d, e, f). In each peculiar analysis, the contribution to the total codon usage variation is indicated, where only the first 10 eigenvalues are represented for comparison. [file 12284_2023_627_MOESM3_ESM.docx]

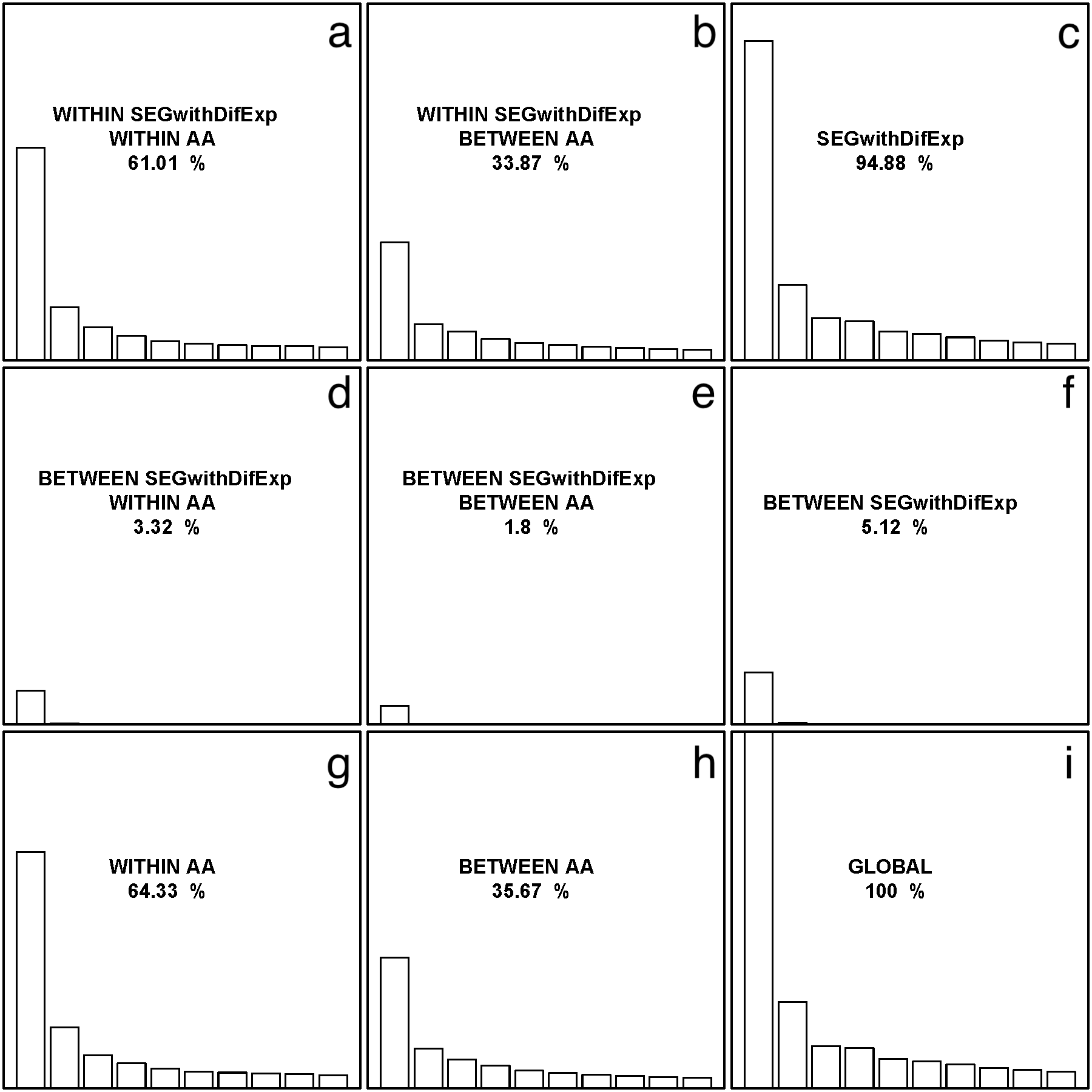


**Supplementary Figure 1. Internal correspondence analysis of single-exon genes with different gene expression level in rice.** The total codon usage variability is decomposed into the synonymous codon usage variability (a, d, g), amino acid usage variability (b, e, h), and variability of within (a, b, c) and between different gene expression groups (d, e, f). The performance of ICA yields nine elementary analyses (a-i). In each peculiar analysis, the contribution to the total codon usage variability is indicated, where only the first 10 eigenvalues are represented for comparison.


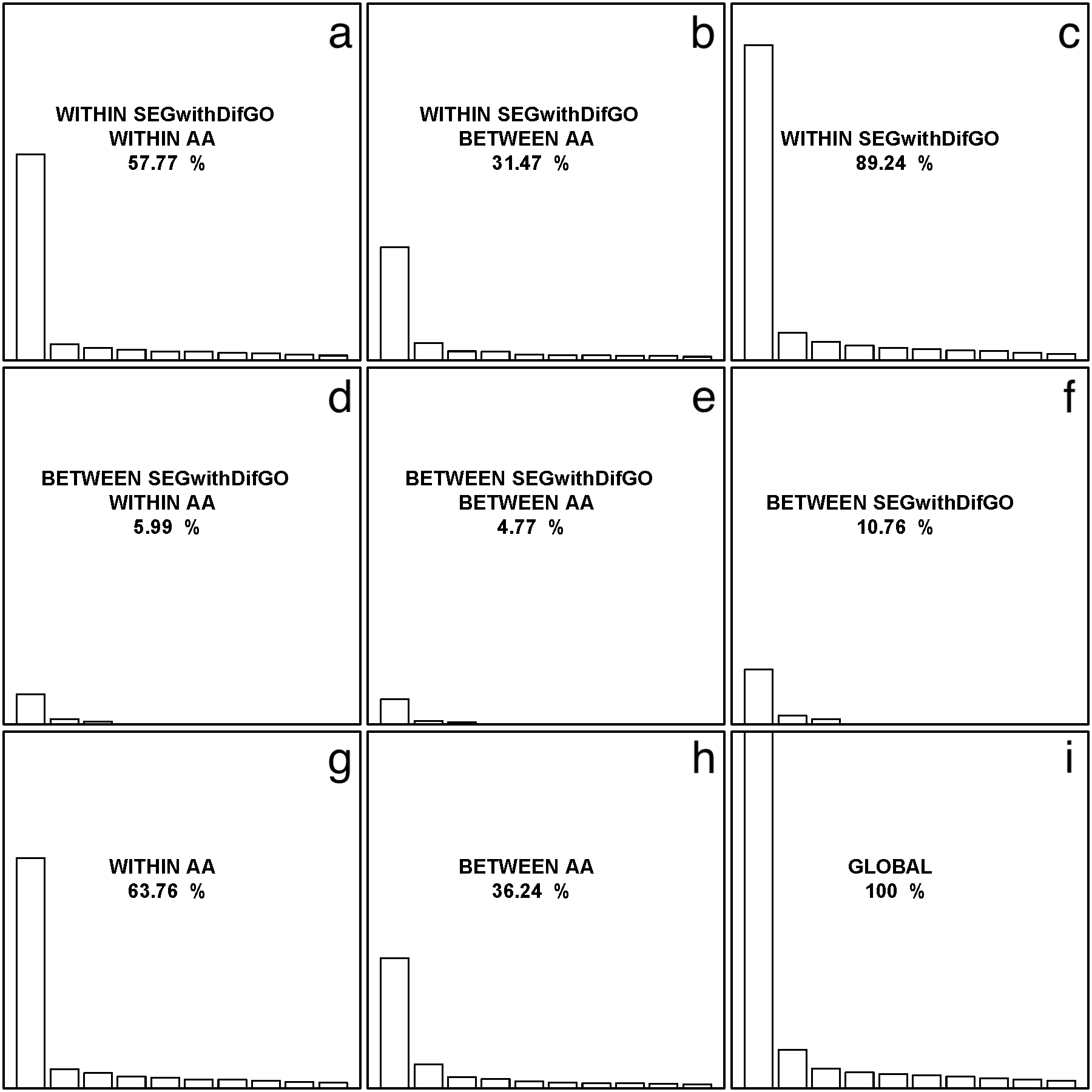


**Supplementary Figure 2. Internal correspondence analysis of single-exon genes with different gene function in rice.** The total codon usage variability is decomposed into the synonymous codon usage variability (a, d, g), amino acid usage variability (b, e, h), and variability of within (a, b, c) and between different gene function groups (d, e, f). The performance of ICA yields nine elementary analyses (a-i). In each peculiar analysis, the contribution to the total codon usage variability is indicated, where only the first 10 eigenvalues are represented for comparison.


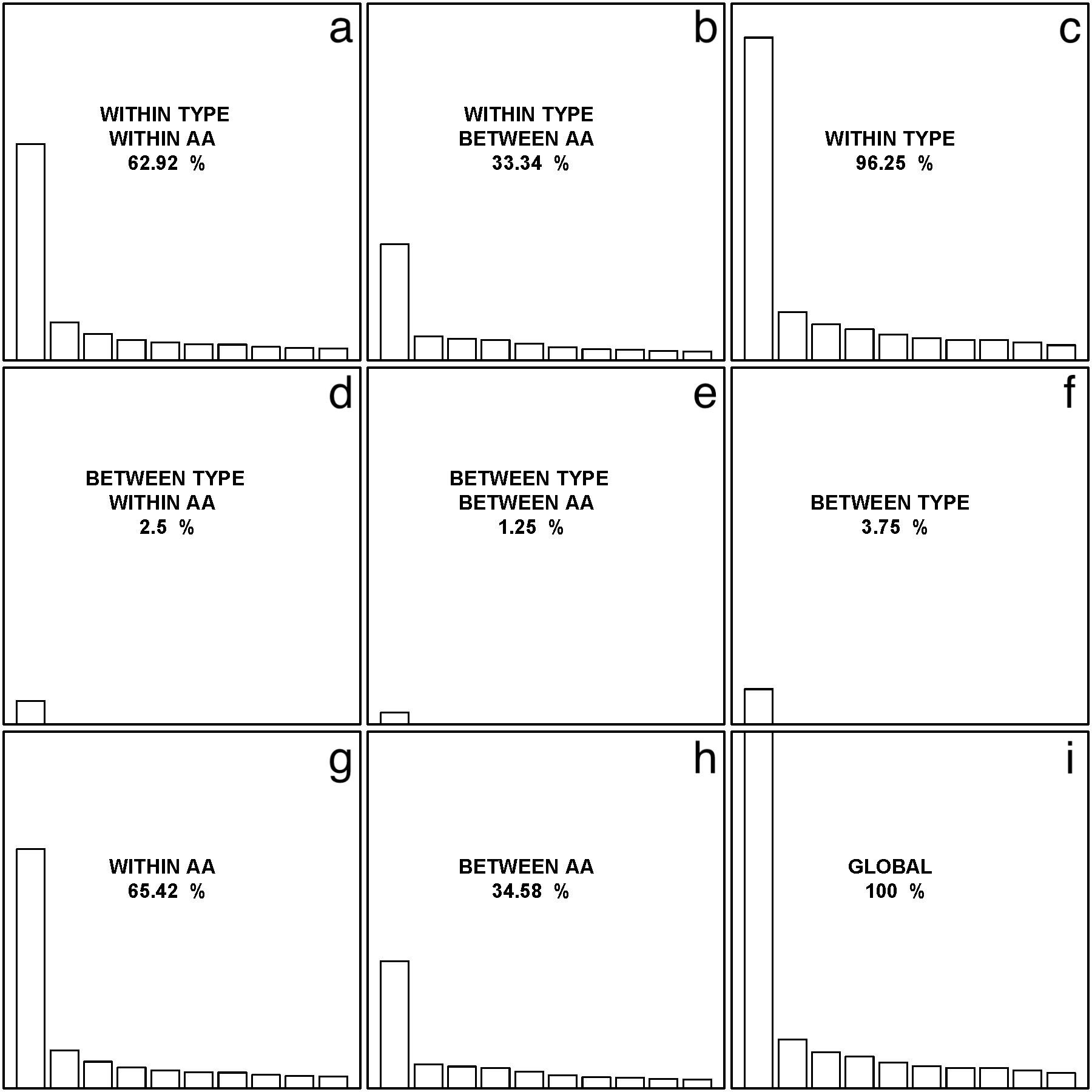


**Supplementary Figure 3. Internal correspondence analysis of single-exon genes and multiple-exon genes in rice**. In this analysis, the 581 paralogous gene pairs of SEGs and MEGs are excluded from the dataset. The total codon usage variability is decomposed into the synonymous codon usage (within-AA) variability (a, d, g), amino acid usage (between-AA) variability (b, e, h), and variability of within (a, b, c) and between gene types (d, e, f). In each peculiar analysis, the contribution to the total codon usage variation is indicated, where only the first 10 eigenvalues are represented for comparison.
